# Supplementary material for: REX1 is the critical target of RNF12 in imprinted X chromosome inactivation in mice
Source: Nat Commun. 2018 Nov 12;9:4752. doi: 10.1038/s41467-018-07060-w (PMC6232137; doi:10.1038/s41467-018-07060-w)
Supplement: Supplementary file 1 — Description of Additional Supplementary Files [file 41467_2018_7060_MOESM1_ESM.pdf]

Description of Additional Supplementary Files

File Name: Supplementary Data 1

Description: SILAC dataset Spreadsheet with the SILAC data referring to Fig. 1 and Supplementary Fig. 1. Table Exp1_3: Heavy (H) SILAC-labelled Rnf12-/- ESCs and light (L) WT ESCs were used for the forward experiment (Exp. 1), and H SILAC-labelled WT ESCs and L Rnf12-/- ESCs for the reverse experiment (Exp 3). Table Exp5_7: H SILAC-labelled MG132- treated ESCs and L DMSOcontrol ESCs were used for the forward experiment (Exp. 5), and H SILAC- labelled DMSOcontrol ESCs and light MG132-treated ESCs for the reverse experiment (Exp.7)
