# Supplementary material for: REX1 is the critical target of RNF12 in imprinted X chromosome inactivation in mice
Source: Nat Commun. 2018 Nov 12;9:4752. doi: 10.1038/s41467-018-07060-w (PMC6232137; doi:10.1038/s41467-018-07060-w)
Supplement: Supplementary file 3 — Supplementary Information [file 41467_2018_7060_MOESM3_ESM.pdf]

Description of Supplementary Files

Title of manuscript: REX1 is the critical target of RNF12 in imprinted X chromosome inactivation in mice

First Author: Gontan and Mira-Bontenbal, et al.

File Name: Supplementary Information

Description: Supplementary Figures, Supplementary Tables and Supplementary References

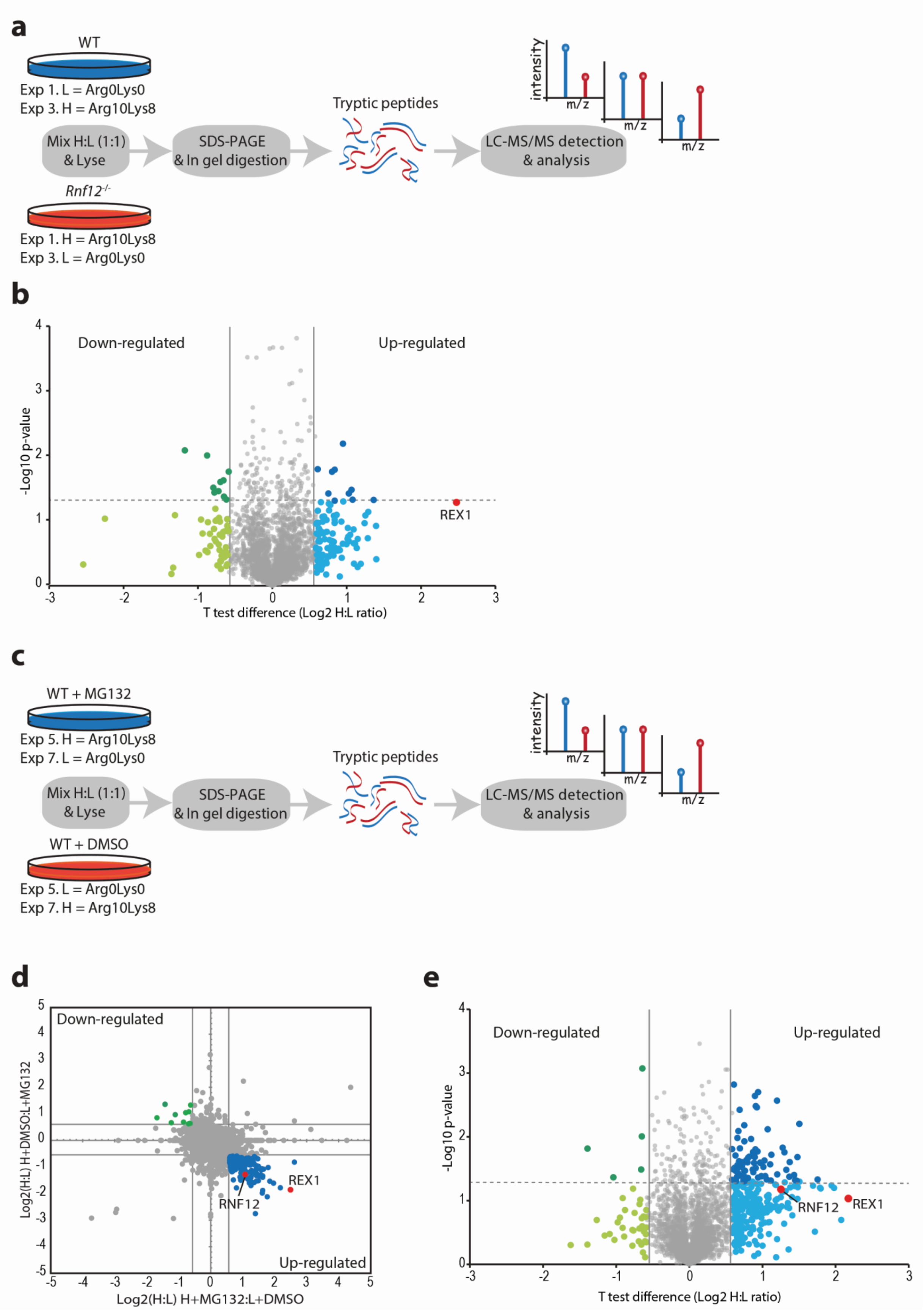
a
WT
Exp 1. L = Arg0Lys0
Exp 3. H = Arg10Lys8
Mix H:L (1:1) & Lyse
SDS-PAGE & In gel digestion
Tryptic peptides
LC-MS/MS detection & analysis
intensity
m/z
Rnf12-/-
Exp 1. H = Arg10Lys8
Exp 3. L = Arg0Lys0
b
Down-regulated
Up-regulated
-Log10 p-value
REX1
T test difference (Log2 H:L ratio)
c
WT + MG132
Exp 5. H = Arg10Lys8
Exp 7. L = Arg0Lys0
Mix H:L (1:1) & Lyse
SDS-PAGE & In gel digestion
Tryptic peptides
LC-MS/MS detection & analysis
WT + DMSO
Exp 5. L = Arg0Lys0
Exp 7. H = Arg10Lys8
d
Down-regulated
Up-regulated
REX1
RNF12
Log2(H:L) H+DMSO:L+MG132
Log2(H:L) H+MG132:L+DMSO
e
Down-regulated
Up-regulated
-Log10 p-value
RNF12
REX1
T test difference (Log2 H:L ratio)

Supplementary Figure 1 | SILAC-based quantification of proteins identified between WT and *RNF12*$^{-/-}$ ESCs and in WT ESCs in the presence or absence of the proteasome inhibitor MG132.

(**a**) Experimental scheme of SILAC experiment to determine RNF12-dependent changes in protein stability. Heavy (H) SILAC-labelled *Rnf12*$^{-/-}$ ESCs and light (L) WT ESCs were used for the forward experiment (Exp. 1), and H SILAC-labelled WT ESCs and L *Rnf12*$^{-/-}$ ESCs for the reverse experiment (Exp 3). (**b**) Volcano plot representing the average log2 fold-change between two biological replicates in (**a**) against the -log 10 (p-values). The horizontal dashed line represents the t-test threshold of significance p-value< 0.05. Proteins up-regulated in *Rnf12*$^{-/-}$ (dark-blue, p < 0.05, > 1.5-fold-change), down-regulated (dark-green) and REX1(red) are indicated. (**c**) Schematic representation of the SILAC experiment to determine proteasome-dependent changes in protein stability. H SILAC-labelled MG132-treated ESCs and L DMSO-control ESCs were used for the forward experiment (Exp. 5), and H SILAC-labelled DMSO-control ESCs and light MG132-treated ESCs for the reverse experiment (Exp.7). (**d**) Scatter plot depicting the correlation of the H:L log2 ratios for the quantified proteins in the SILAC experiment between two biological replicates in (**c**). RNF12 and REX1 are stabilized in the presence of MG132 (red). Blue dots indicate up-regulated proteins (log2 ratios >0.585 and log2 ratios <-0.585 on the *x* and *y*-axes respectively) and green dots indicate depleted proteins (log2 ratios <-0.585 and log2 ratios >0.585 on the *x* and *y* axes respectively). (**e**) Volcano plot representing the average log2 fold-change between two biological replicates in (**c**) against the -log 10 (p-values). The horizontal dashed line represents the t-test threshold of significance p-value< 0.05. Proteins up-regulated in the presence of MG132 (dark-blue, p < 0.05, > 1.5-fold-change), down-regulated (dark-green) and REX1 and RNF12 (red) are indicated.

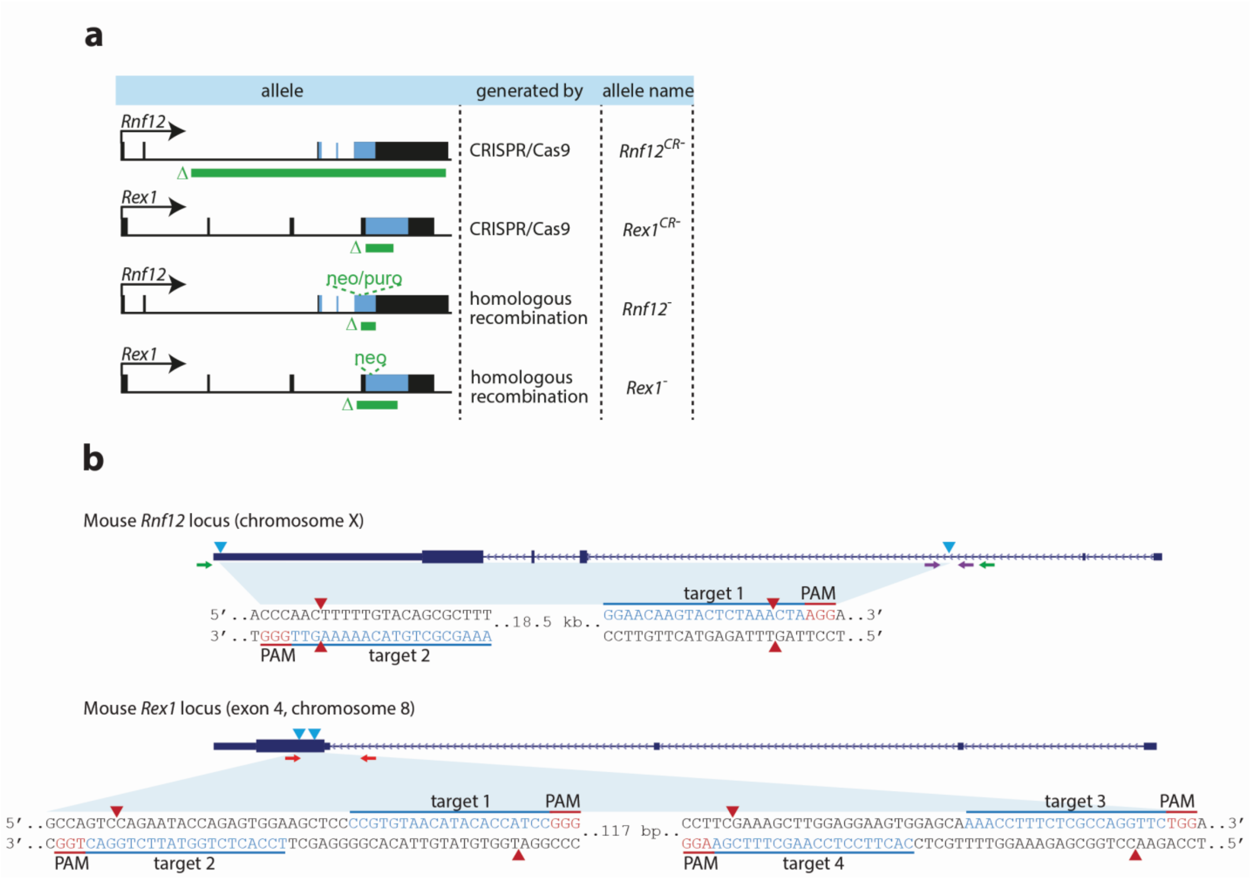

Supplementary Figure 2 | Summary of different *Rnf12* and *Rex1* knockout ES cell lines and mice in this study. (**a**) Schematic representation of the different ESC lines and mice generated in this study. The *Rnf12*$^{CR-}$ allele was generated with two CRISPR/Cas9 nucleases to delete the entire coding region of *Rnf12*. The *Rex1*$^{CR-}$ allele was generated with four CRISPR/Cas9 nickases to delete part of the *Rex1* coding region. The *Rnf12*$^{-}$ allele was generated by the insertion of a drug-resistance cassette (Neomycin, Neo or Puromycin, Puro) via homologous recombination disrupting the catalytic Ring finger domain. The *Rex1*$^{-}$ allele was generated by the insertion of a Neo resistance cassette via homologous recombination and deletion of a 720bp DNA fragment including the ATG starting codon. (**b**) Representation of the mouse *Rnf12* locus with the CRISPR/Cas9 nuclease target sequences (upper panel). Depiction of the mouse *Rex1* locus with the CRISPR/Cas9 nickase target sequences (lower panel). Corresponding green, purple and red arrows indicate location of genotyping primers to confirm the deletion (Supplementary Table 1). Red triangles indicate cutting sites.

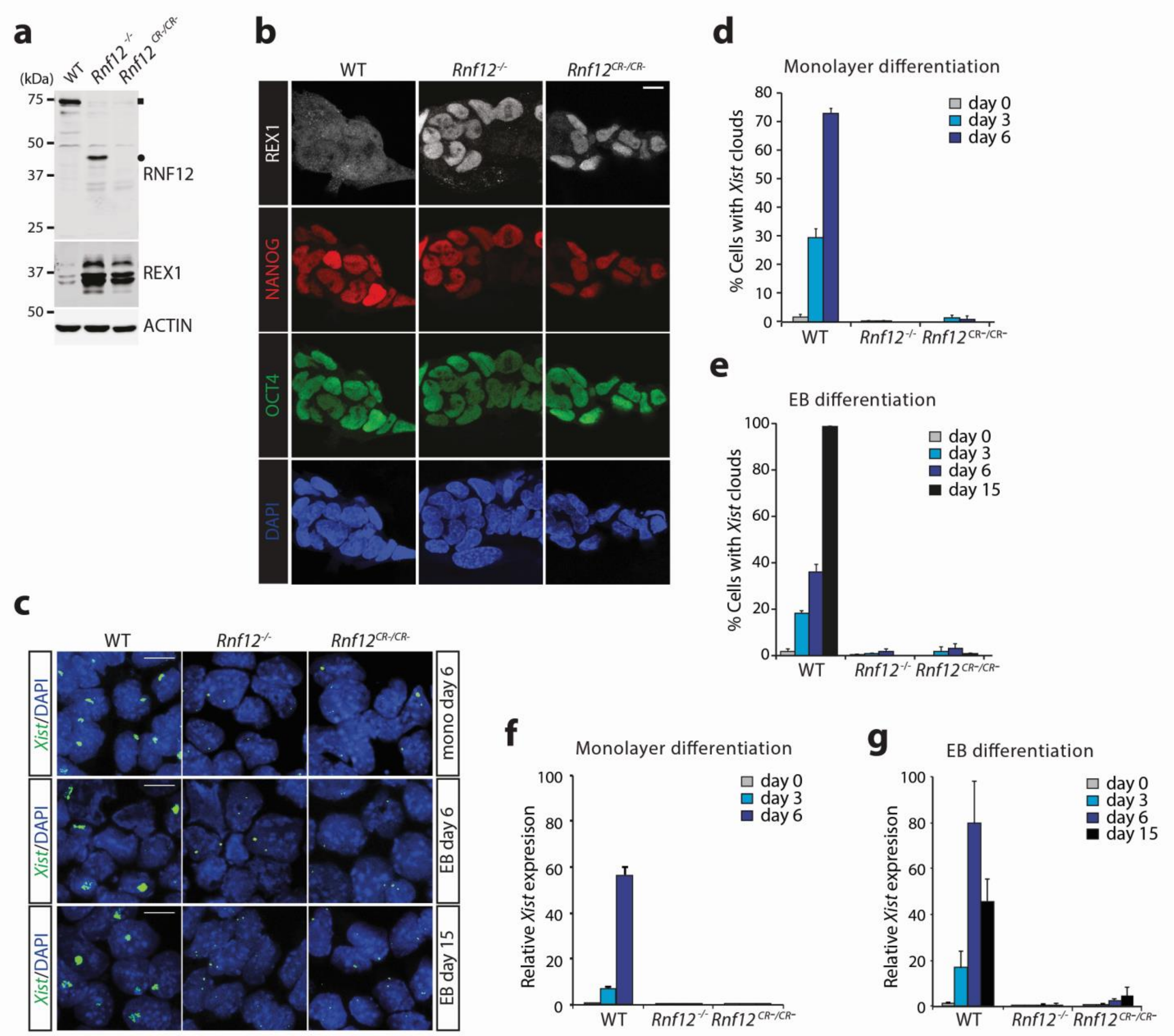

Supplementary Figure 3 | *Rnf12*$^{CR-/CR-}$ and *Rnf12*$^{-/-}$ ESCs do not undergo rXCI *in vitro*. (**a**) Nuclear extracts of WT, *Rnf12*$^{-/-}$ and *Rnf12*$^{CR-/CR-}$ ESCs were immunoblotted with RNF12 and REX1 antibodies. ACTIN was used as loading control (running positions of WT RNF12 and the RNF12 333 aa fragment are indicated with a black square and a black circle, respectively). Uncropped WB images are found in Supplementary Fig. 10c. (**b**) Immunohistochemistry of REX1 (grey), NANOG (red), OCT4 (green) and DNA (DAPI, blue) of WT, *Rnf12*$^{-/-}$ and *Rnf12*$^{CR-/CR-}$ undifferentiated ESCs. Scale bar: 20 μm. (**c**) *Xist* RNA-FISH (FITC) analysis on WT, *Rnf12*$^{-/-}$, *Rnf12*$^{CR-/CR-}$ ESCs at day 6 of differentiation (monolayer and EB differentiation), and day 15 of EB differentiation. DNA was stained by DAPI (blue). Scale bar: 20 μm. (**d,e**) Quantification of cell with *Xist* clouds in WT, *Rnf12*$^{-/-}$, *Rnf12*$^{CR-/CR-}$ ESCs at day 0, 3 and 6 of a monolayer (**d**) or EB (**e**) differentiation (average expression ± s.d., n=3 biological replicates). (**f,g**) QPCR analysis of *Xist* expression in WT, *Rnf12*$^{-/-}$, *Rnf12*$^{CR-/CR-}$ ESCs at day 0, 3 and 6 of a monolayer (**f**) or EB (**g**) differentiation (average expression ± s.d., n=3 biological replicates).

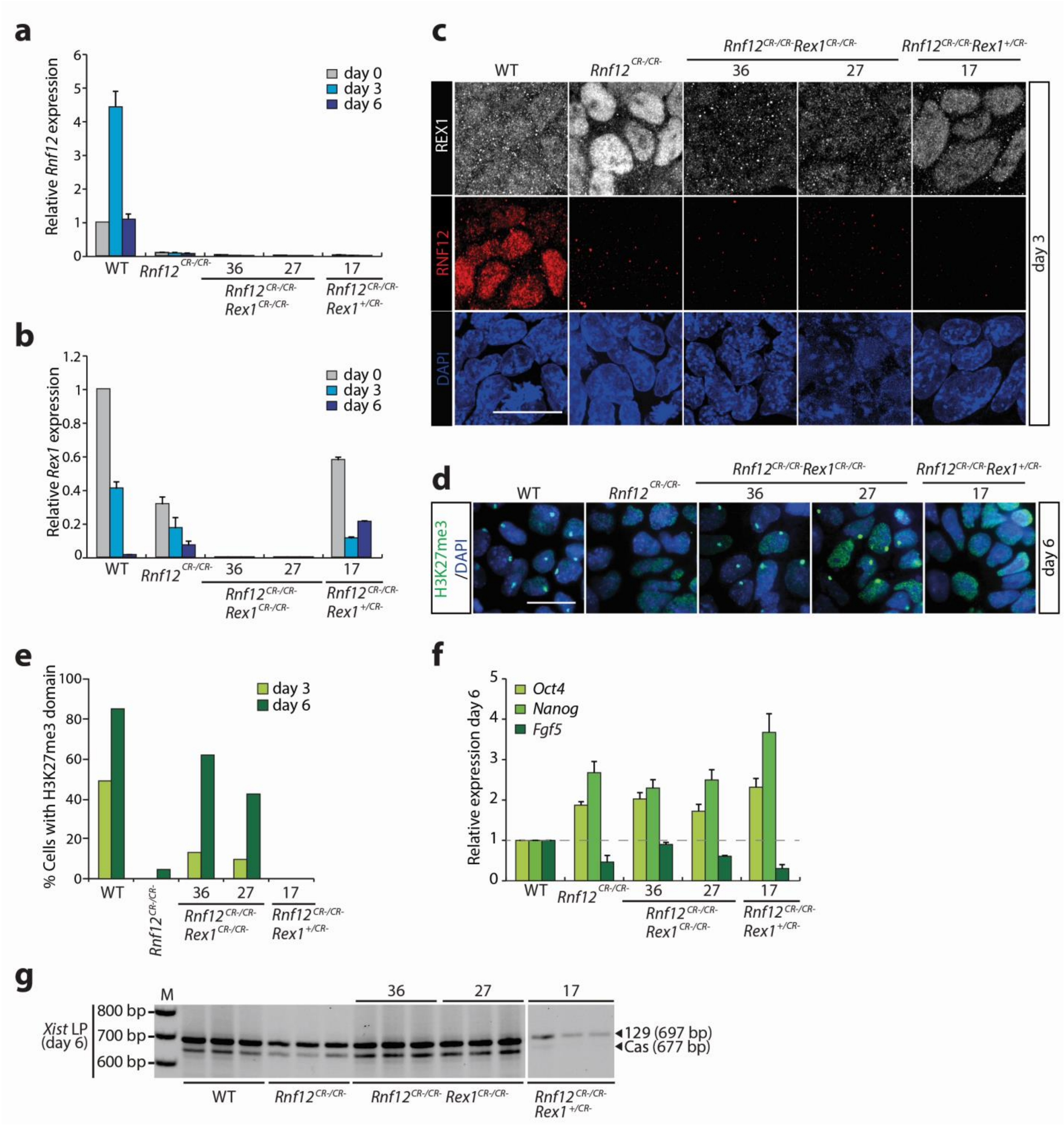

Supplementary Figure 4 | *Rnf12*$^{CR-/CR-}$:*Rex1*$^{CR-/CR-}$ ESCs display rescue of the rXCI phenotype observed in *Rnf12*$^{CR-CR/-}$ ESCs. (**a**,**b**) QPCR analysis of *Rnf12* (**a**) and *Rex1* (**b**) expression in undifferentiated, day 3 and day 6 differentiated WT, *Rnf12*$^{CR-/CR-}$, *Rnf12*$^{CR-/CR-}$:*Rex1*$^{CR-/CR-}$ (clones 36 and 27) and *Rnf12*$^{CR-/CR-}$:*Rex1*$^{+/CR-}$ (clone 17) ESCs (average expression ± s.d., n=3 biological replicates). (**c**) Immunohistochemistry of REX1 (grey), RNF12 (red) and DNA (DAPI, blue) of WT, *Rnf12*$^{CR-/CR-}$, *Rnf12*$^{CR-/CR-}$:*Rex1*$^{CR-/CR-}$ (clones 36 and 27), and *Rnf12*$^{CR-/CR-}$:*Rex1*$^{+/CR-}$ (clone 17) ESCs at day 3 of monolayer differentiation. Scale bars: 20 µm. (**d**) Representative images of an H3K27me3 (Xi marker, green) immunostaining of WT, *Rnf12*$^{CR-/CR-}$, *Rnf12*$^{CR-/CR-}$:*Rex1*$^{CR-/CR-}$ (clones 36 and 27) and *Rnf12*$^{CR-/CR-}$:*Rex1*$^{+/CR-}$

(clone 17) ESCs at day 6 of a monolayer differentiation. DNA was stained by DAPI (blue). Scale bar: 20 µm. (**e**) Quantification of cells with H3K27me3 domains in WT, *Rnf12*$^{CR-/CR-}$, *Rnf12*$^{CR-/CR-}$:*Rex1*$^{CR-/CR-}$ (clones 36 and 27) and *Rnf12*$^{CR-/CR-}$:*Rex1*$^{+/CR-}$ (clone 17) ESCs at day 3 and day 6 of a monolayer differentiation in (**d**). (**f**) QPCR analysis of pluripotency markers *Oct4*, *Nanog* and differentiation marker *Fgf5* in WT, *Rnf12*$^{CR-/CR-}$, , *Rnf12*$^{CR-/CR-}$:*Rex1*$^{CR-/CR-}$ (clones 36 and 27), and *Rnf12*$^{CR-/CR-}$:*Rex1*$^{+/CR-}$ (clone 17) ESCs at day6 of a monolayer differentiation (average expression ± s.d., n=3 biological replicates). (**g**) Allele-specific *Xist* RNA expression analysis in WT, *Rnf12*$^{CR-/CR-}$, *Rnf12*$^{CR-/CR-}$:*Rex1*$^{CR-/CR-}$ (clones 36 and 27), and *Rnf12*$^{CR-/CR-}$:*Rex1*$^{+/CR-}$ (clone 17) ESCs at day 6 of a monolayer differentiation (n=3). M, is a 100 bp DNA ladder marker (NEB).

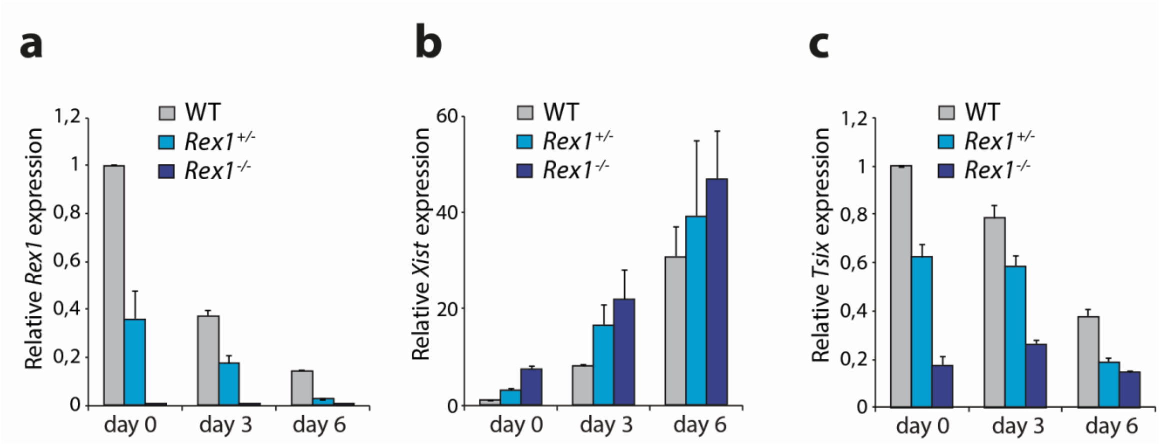

Supplementary Figure 5 | Differentiating *Rex1*$^{-/+}$ and *Rex1*$^{-/-}$ ESCs show increased *Xist* expression and decreased *Tsix* expression compared to WT cells. (**a,b,c**) *Rex1* (**a**), *Xist* (**b**) and *Tsix* (**c**) qPCR analysis at day 0, day 3 and day 6 of a monolayer differentiation of WT, *Rex1*$^{-/+}$ and *Rex1*$^{-/-}$ ESCs (average expression ± s.d., n=3 biological replicates).

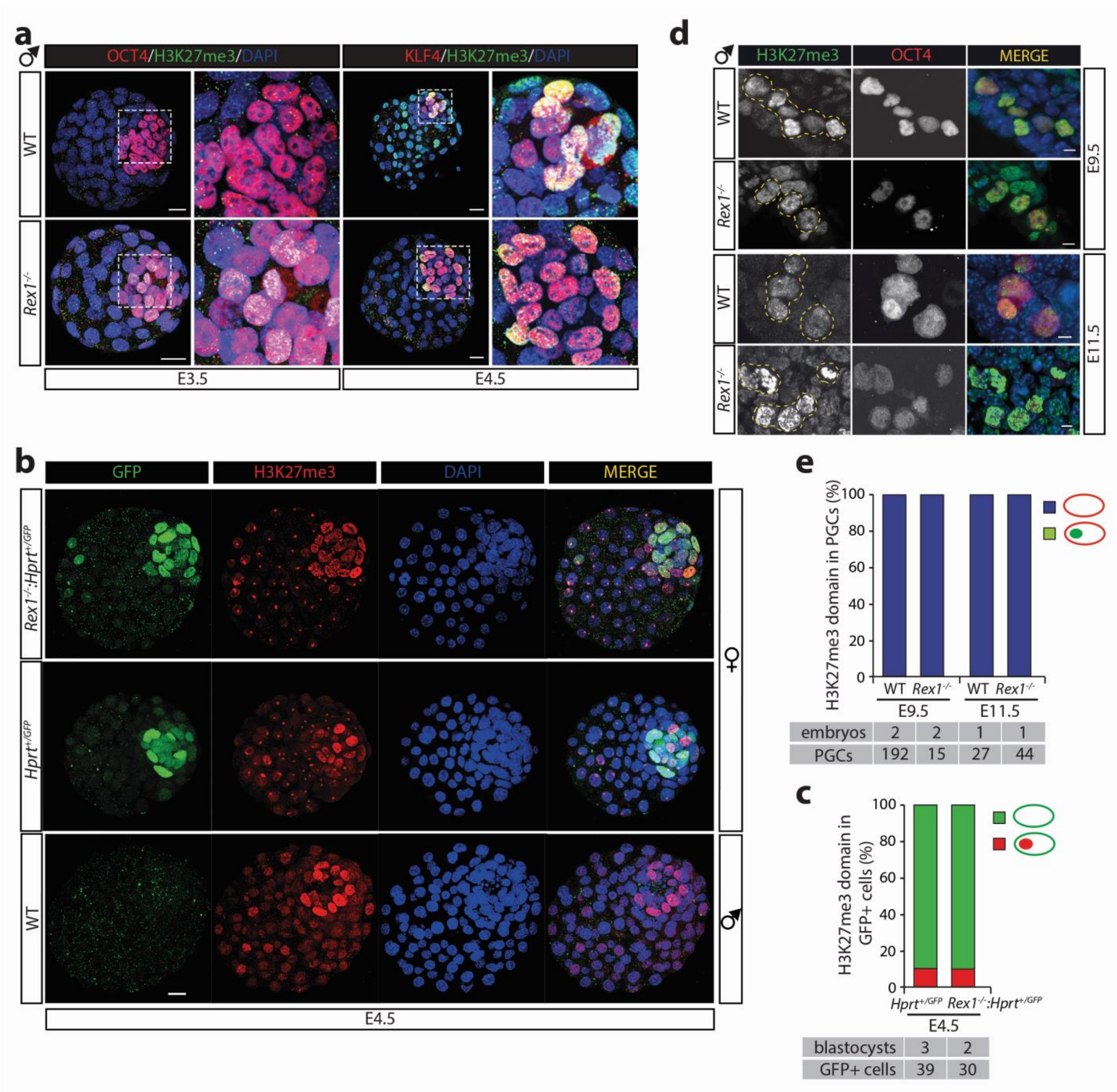

Supplementary Figure 6 | XCR is not compromised in Rex1 mutant mice. (**a**) Representative *Z*-stack projections of WT and *Rex1*$^{-/-}$ male E3.5 and E4.5 blastocysts immunostained for H3K27me3 (Xi marker, green) and the lineage markers OCT4 (E3.5 ICM, red, left panels) and KLF4 (E4.5 epiblast, red, right panels). Whole embryo and ICM/epiblast higher magnification for each embryo are shown (white boxes). DNA was stained by DAPI (blue). Scale bars: 20 μm. (**b**) Representative *Z*-stack projections of *Rex1*$^{-/-}$:*Hprt*$^{+/GFP}$ and *Hprt*$^{+/GFP}$ female and WT male E4.5 blastocysts immunostained for H3K27me3 (Xi marker, red) and GFP (marker for reactivation of imprinted paternal Xi in epiblast cells, green). Scale bars: 20 μm. (**c**) Quantification of the number of GFP-positive cells with and without H3K27me3 domains in *Hprt*$^{+/GFP}$ and *Rex1*$^{-/-}$:*Hprt*$^{+/GFP}$ female E4.5 blastocysts. The number of blastocysts and cells counted is indicated. (**d**) Representative paraffin sections of male WT and *Rex1*$^{-/-}$ E9.5 embryo hindguts and E11.5 male

embryo trunks immunostained for OCT4 (PGC marker, red) and H3K27me3 (Xi marker, green). Representative PGCs are marked with yellow dashed lines. Scale bars: 5 µm. (**e**) Quantification of cells with an H3K27me3 domain in male WT and *Rex1*$^{-/-}$ PGCs at E9.5 and E11.5. Number of embryos and PGCs analysed are indicated.

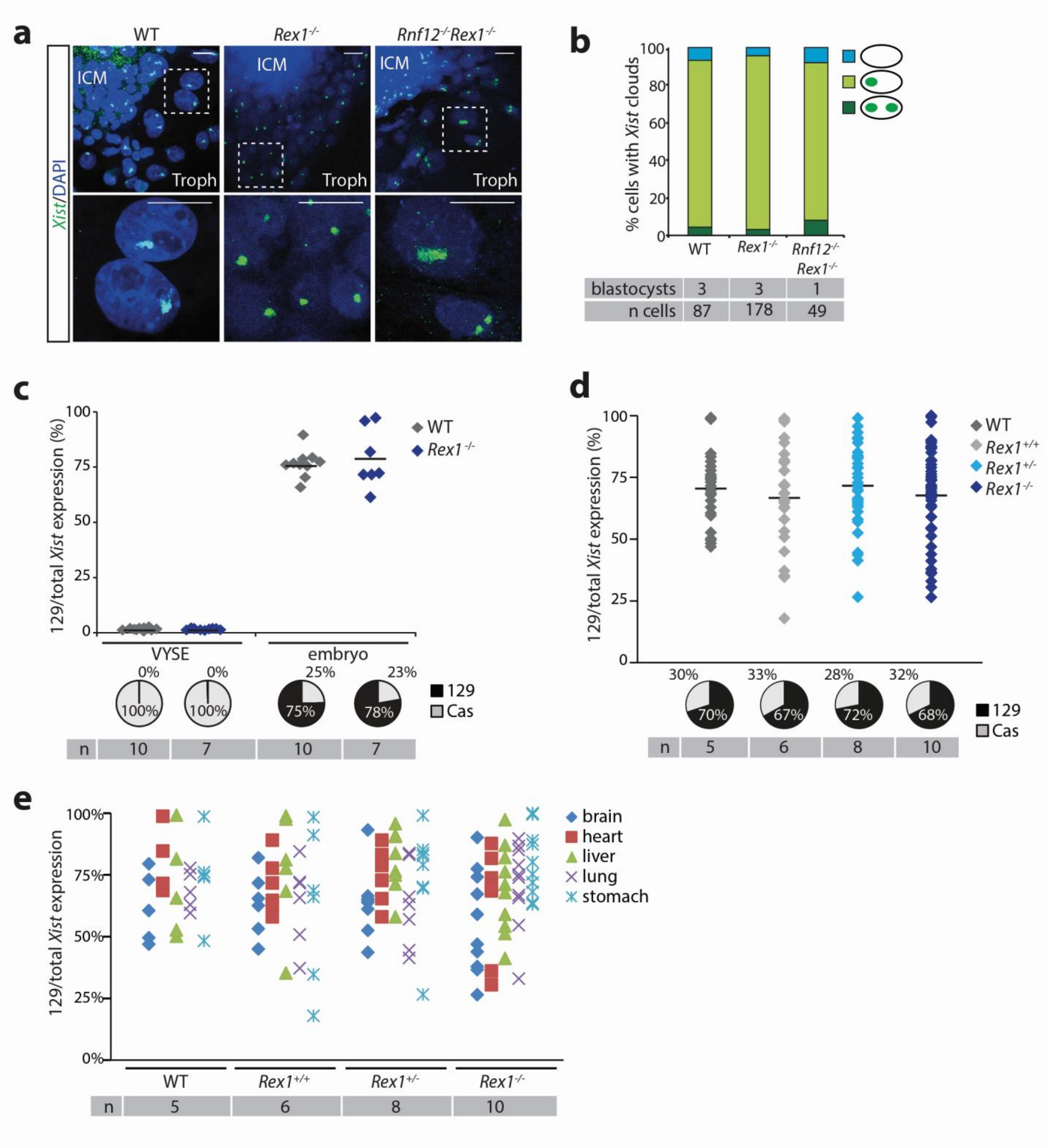

Supplementary Figure 7 | iXCI and rXCI are not compromised in *Rex1* mutant mice. (**a**) *Xist* RNA-FISH (FITC) analysis on female WT, *Rex1*$^{-/-}$ and *Rnf12*$^{-/-}$:*Rex1*$^{-/-}$ E3.5 blastocyst outgrowths. White boxes indicate selected trophoblast (Troph) cells shown below at a higher magnification. Scale bars: 20 µm. (**b**) Quantification of Xist-positive trophoblast cells in female WT, *Rex1*$^{-/-}$ and *Rnf12*$^{-/-}$:*Rex1*$^{-/-}$ blastocyst outgrowths from (**a**). Number of embryos and cells analysed are indicated. (**c**) Quantification of the 129 allelic expression ratio of *Xist* in E11.5 WT and *Rex1*$^{-/-}$ female embryos and corresponding VYSE in Fig. 3f. Pie charts show the average ratio of allelic 129 (black) and cas (grey) *Xist* expression. The number of

mice analysed is indicated. Horizontal bars overlaying dot clusters represent their average. (**d**) Quantification as in (**c**) but in WT, *Rex1*$^{+/+}$ (mice obtained from *Rex1*$^{+/-}$ crosses), *Rex1*$^{+/-}$, *Rex1*$^{-/-}$ organs of 4-weeks-old female mice in Fig. 3h, where each dot represents the ratio of 129 allelic *Xist* expression in a single organ (heart, liver, stomach, brain, lung). The number of mice analysed is indicated. Horizontal bars overlaying dot clusters represent their average. (**e**) Another representation of (**d**) where the *Xist* expression ratio is plotted per organ of each individual mouse.

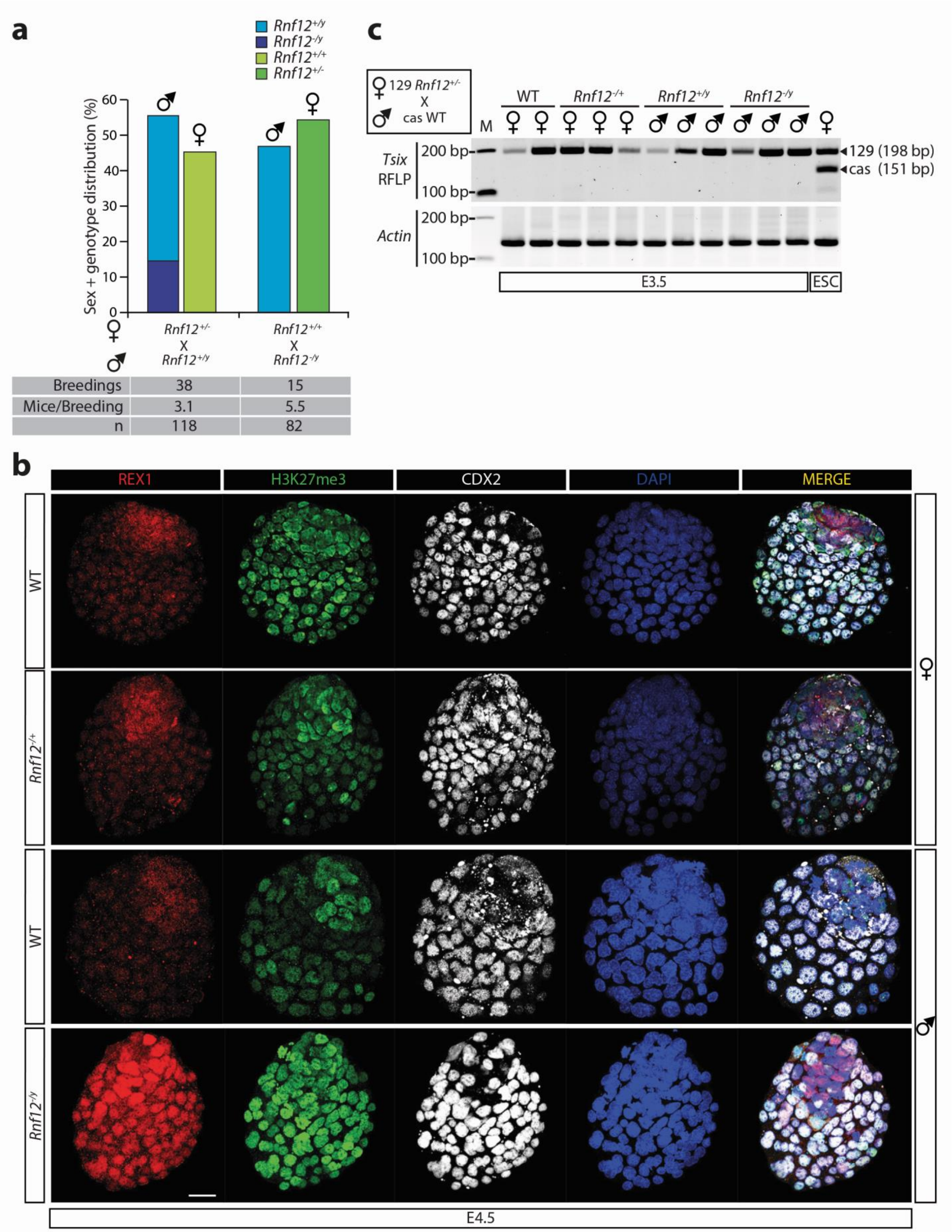

Supplementary Figure 8 | *Rnf12*$^{-/+}$ embryos do not show REX1 accumulation. (**a**) Sex and genotype distribution from different *Rnf12* mutant crossings in a Cast/EiJ background. Number of breedings,

number of mice per breeding and total number of mice are indicated. Note that no female embryos were born with a maternally transmitted Rnf12 deleted allele. (**b**) Representative *Z*-stack projections of WT and *Rnf12*$^{-/+}$ female and WT and *Rnf12*$^{-/Y}$ male E4.5 embryos immunostained for REX1 (red), H3K27me3 (Xi marker, green) and the trophectoderm marker CDX2 (grey). Female *Rnf12*$^{-/+}$ blastocysts presented few trophoblast cells with a H3K27me3 domain (imprinted Xi) and no detectable accumulation of REX1 protein. All embryos were generated by crossing *Rnf12*$^{+/-}$ females with WT males. Scale bars: 20 µm. (**c**) *Tsix* allele-specific RNA expression analyses from E3.5 blastocysts WT and *Rnf12*$^{-/+}$ female and WT and *Rnf12*$^{-/Y}$ male blastocysts generated by crossing 129 *Rnf12*$^{+/-}$ females with cas WT males. M, is a 100 bp DNA ladder marker (NEB). *Actin* is a positive control to confirm the presence of cDNA.

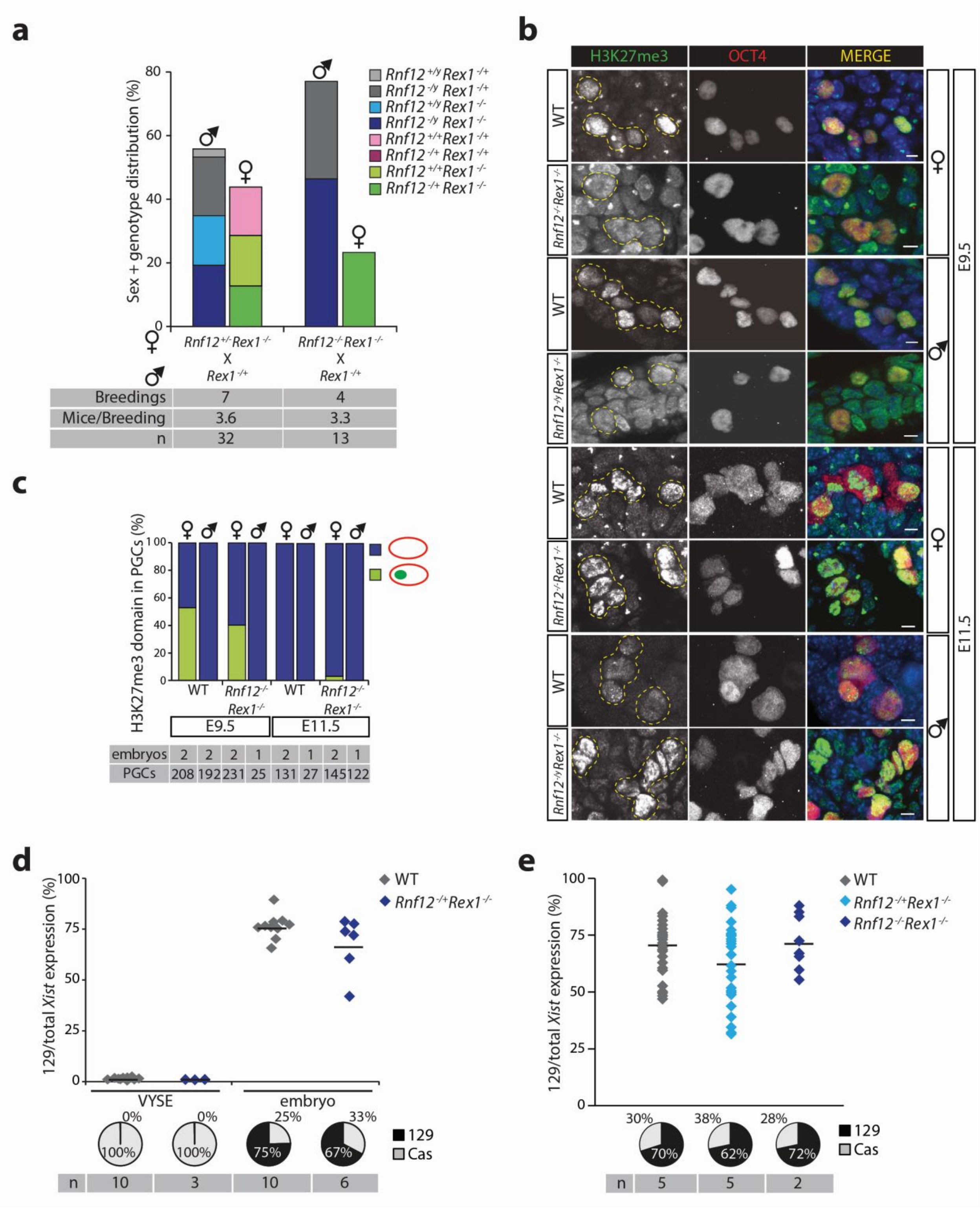

Supplementary Figure 9 | *Rnf12$^{-/-}$:Rex1$^{-/-}$* double knockout mice are viable and have normal iXCI and rXCI. (**a**) Sex and genotype distribution from different *Rnf12* mutant crossings in an *Rex1* mutant background. Number of breedings, number of mice per breeding and total number of mice are indicated.

Note that no female embryos were born with a maternally transmitted *Rnf12* deleted allele in a *Rex1*$^{-/+}$ background. (**b**) Paraffin sections of female and male WT and *Rnf12*$^{-/-}$:*Rex1*$^{-/-}$ *or Rnf12*$^{-/y:}$*Rex1*$^{-/-}$ E9.5 embryo hindguts and E11.5 embryo trunks immunostained for OCT4 (PGCs marker, red) and H3K27me3 (Xi marker, green H3K27me3 domains are present in female somatic cells and in some female E9.5 PGCs, while they are lost in E11.5 PGCs (XCR). Representative PGCs are marked with yellow dashed circles. WT samples are same control samples as in Fig. 3c. Scale bars: 5 µm. (**c**) Percentages of cells with a H3K27me3 domain (green) in female and male WT and *Rnf12*$^{-/-}$:*Rex1*$^{-/-}$ E9.5 and E11.5 PGCs. WT samples are same control samples as in Fig. 3d. Number of embryos and PGCs analysed are indicated. No difference was seen in the percentage of PGCs with a H3K27me3 domain in the WT versus KO mouse embryos. (**d**) Quantification of the 129 allelic *Xist* expression in E11.5 female WT and *Rnf12*$^{-/+}$:*Rex1*$^{-/-}$ embryos and associated VYSE in Fig. 5d. WT samples are same control samples as in Fig. 3f. Pie charts show the relative *Xist* expression emanating from the 129 (black) and cas (grey) allele. The number of analysed mice is indicated. Horizontal bars overlaying dot clusters represent their average. (**e**) Quantification of the 129 allelic ratio of *Xist* expression in several WT and *Rnf12*$^{-/-}$:*Rex1*$^{-/-}$ organs from 4-weeks-old female mice in Fig. 5f. Each dot represents the 129 allelic *Xist* expression in a single organ (heart, liver, stomach, brain, lung). WT samples are same control samples as in Fig. 3h. Pie charts show the ratio of 129 (black) and cas (grey) allelic origin of *Xist* expression. The number of mice analysed is indicated. Horizontal bars overlaying dot clusters represent their average.

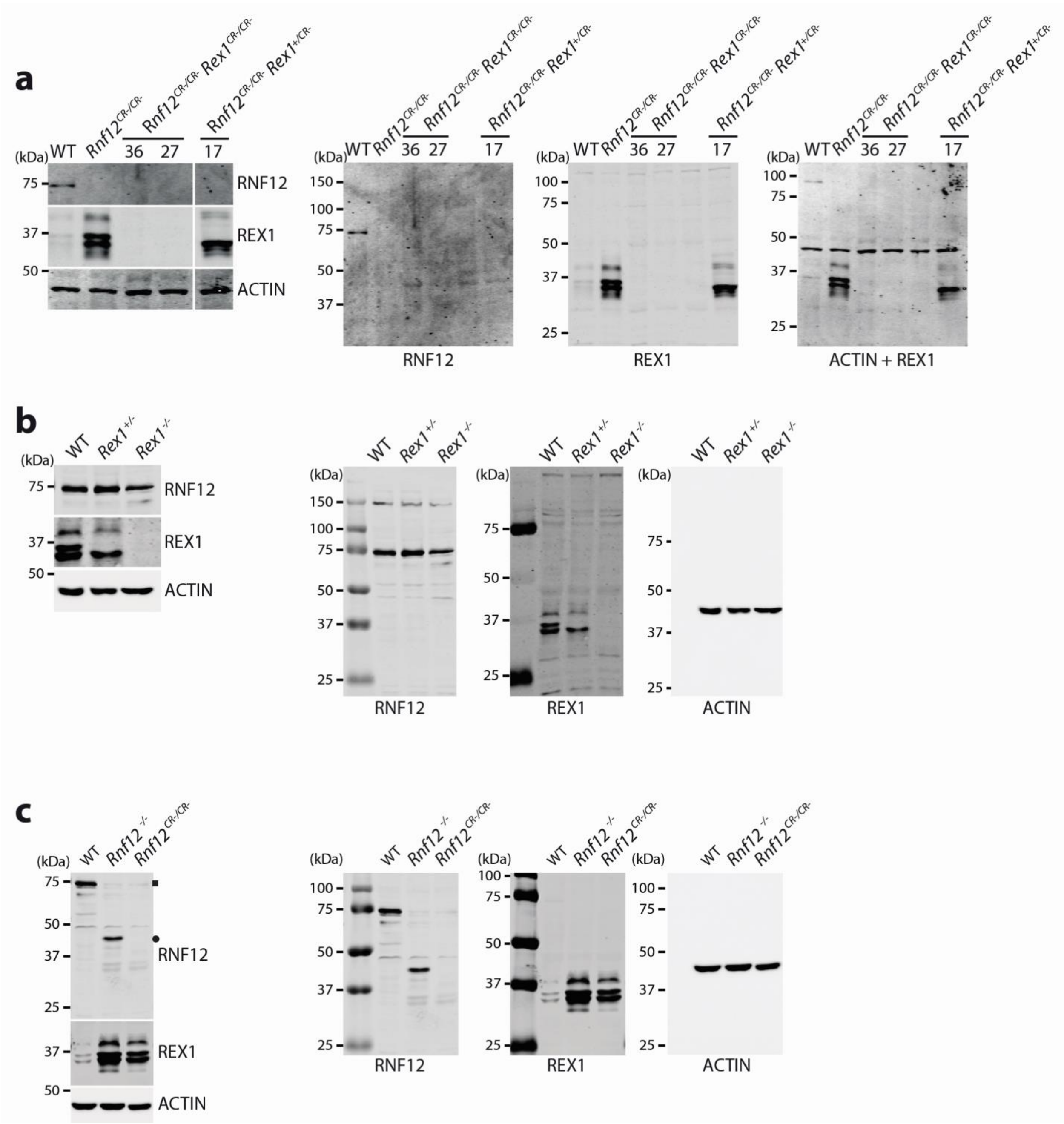

Supplementary Figure 10 | Uncropped Western Blot images. (**a**) Uncropped RNF12 (second panel), REX1 (third panel) and ACTIN (right panel) Western Blot images of Fig. 1c (left panel). (**b**) Uncropped RNF12 (second panel), REX1 (third panel) and ACTIN (right panel) Western Blot images of Fig. 2c (left panel). (**c**) Uncropped RNF12 (second panel), REX1 (third panel) and ACTIN (right panel) Western Blot images of Supplementary Fig. 3a (left panel), (running positions of WT RNF12 and the RNF12 333 aa fragment are indicated with a black square and a black circle, respectively).

**Supplementary Table 1 | List of the PCR primer sets used in this research.**		
	**FW primer**	**RV primer**
**Primers used for constructing the *Rex1* KO mice**		
Rex1 5' arm generation PCR	ggtacccatccccttcaatagcacatatag	gctagcagttcccttctcgagtcttctttgg
Rex1 3' arm generation PCR	gctagcgagagaacacctttgacaaaacc	cggctcattatctatttctgtcc
Rex1 3' arm integration check	caaagttggccattctttagg	ctggcactctgtcgataccc
Rex1 5' arm integration check	acatttccccgaaaagtgc	gtgtagaggtctttgagccactt
Rex1 RFLP (XmnI)	tcgataagacaccacagtacaca	taagatgctttgacgtcttcct
Rex1 genotyping	acatttccccgaaaagtgc	taagatgctttgacgtcttcct
X chr RFLP (Pf1M1)	aattcacgttctcctctttcact	tccccaatta aaggtgttga
SRY	gtggtcccgtggtgagag	ttttgttgaggcaactgcag
Sex[1]	gatgatttgagtggaaatgtgaggta	cttatgtttataggcatgcaccatgta
**Primers used for genotyping the *Rnf12* KO mice and ESCs**		
Rnf12 WT	gccttcgaacatctctgagc	gagccggactaatccaaaca
Rnf12 KO B6	acatttccccgaaaagtgc	gagccggactaatccaaaca
Rnf12 KO CAS	gccttcgaacatctctgagc	agcaaaaacaggaaggcaaa
PCR over deletion	ctgcagtgaatcctccttga	tgaacaaattgtgtgcatgg
ScaI_RFLP	gactgtgggggctactcaga	gatcccctggaaccaaagtt

**Supplementary Table 2 | RT-PCR primers**		
**Gene**	**FW primer**	**RV primer**
Xist LP	gcaacaacgaattagacaacac	actgggtcttcagcatga
		actgggtcttcagcgtga
G6pdx<br>*RFLP*[2]*(ScrFI)*	ggagtgatgaactcagggaagc	atgtagctgggtttactggtgg
Mecp2<br>*RFLP (DdeI)*	catggtagctgggatgttagg	gcaatcaattctactttagagcg
Tsix_A<br>*RFLP*	gccaaattcagcaatactacctc	ggaaataaacggaacgcagtacc
Tsix_B<br>*RFLP (MnlI)*[3]	ggtaacaattttcccgccatgtg	ggaaataaacggaacgcagtacc

**Supplementary Table 3 | QPCR primers**		
**Gene**	**FW primer**	**RV primer**
Xist	ggatcctgcttgaactactgc	caggcaatccttcttcttgag
Tsix	Tgaccagtacctcgcaagttc	Ctaagagcacctggctccac
Rex1	ctaaagcaagacgaggcaag	agaatgggttcggaaaactc
Rnf12	ggtccaccaccacagagc	tgaccacttcttgttgtatttcc
Oct4	ccccaatgccgtgaagttg	tcagcagcttggcaaactgtt
Nanog	aggatgaagtgcaagcggtg	tgctgagcccttctgaatcag
Fgf5	gctgtgtctcaggggattgt	cactctcggcctgtcttttc
Actin	actattggcaacgagcggttc	agaggtctttacggatgtcaacg

Supplementary References

1. McFarlane, L., Truong, V., Palmer, J. S. & Wilhelm, D. Novel PCR assay for determining the genetic sex of mice. *Sex Dev* **7,** 207–211 (2013).
2. Minkovsky, A. *et al.* The pluripotency factor-bound intron 1 of Xist is dispensable for X chromosome inactivation and reactivation in vitro and in vivo. *Cell Rep* **3,** 905–918 (2013).
3. Lee, J. T. Disruption of imprinted X inactivation by parent-of-origin effects at Tsix. *Cell* **103,** 17–27 (2000).
